# Time lapse imaging analysis of the effect of ER stress modulators on apoptotic cell assessed by caspase3/7 activation in NG108-15 cells

**DOI:** 10.1016/j.dib.2015.11.030

**Published:** 2015-11-21

**Authors:** Ayako Saito, Kei Suga, Risa Ono-Nakagawa, Masumi Sanada, Kimio Akagawa

**Affiliations:** Department of Cell Physiology, Kyorin University School of Medicine, Mitaka, Tokyo 181-8611, Japan

## Abstract

This paper reports the data from the long term time lapse imaging of neuronal cell line NG108-15 that were treated with apoptosis inducer or various ER stress inducers. Use of the fluorescent reporter for activated caspase3/7 in combination with the conventional light microscope allowed us to investigate the time course of apoptosis induction at the single cell level. Quantitative as well as qualitative data are presented here to show the effect of two different ER stress modulating chemical compounds on caspase3/7-dependent apoptosis in neuronal cell line NG108-15 cells. Additional results and interpretation of our data concerning ER stress and apoptosis in NG108-15 cells can be found in Suga et al. (2015) [1] and in Suga et al. (2015) [2].

**Specifications Table**

**Table:** 

Subject area	*Biology*
More specific subject area	*Apoptosis, ER Stress response, Caspase, Cell death*
Type of data	*text file, graph, figure*
How data was acquired	*Microscope, time lapse imaging, luminometer*
Data format	*analyzed*
Experimental factors	*NG108-15 cells were treated with apoptosis inducer or different ER stress inducer together with chemical compounds that modulated ER stress and viability assays.*
Experimental features	*ER stress or apoptosis induced cells were processed for time lapse imaging*
Data source location	*Kyorin University School of Medicine, Tokyo, Japan*
Data accessibility	*All data are provided in this article*

**Value of the data**

• The results provided here may inform many researchers that are investigating the relationship of ER stress, apoptosis, and neuronal cell death.
• The data provides important information on the use of various ER stress inducers and modulators in neuronal cell lines.
• Chemical chaperone 4-phenylbutyrate (PBA) showed alleviation of caspase3/7 dependent apoptosis induced by ER stress in NG108-15 cells.
• Data of the time course and the population of the cells showing caspase3/7 dependent apoptosis presented here can serve as a benchmark to the researchers who investigate the effect of ER stress using end-point assays.

## 1 Data

We recently showed that syntaxin5 (Syx5) protein, one of the ER–Golgi SNARE proteins, is upregulated by ER stress, but downregulated by caspase3-dependent apoptosis in neuronal cells [1], [2], [3], [4]. Importantly, caspase3 has been identified as a key mediator of neuronal cell death [5], and it has been implicated that caspase3 is a potential target for pharmacological therapy during early stages of Alzheimer׳s disease [5]. We showed that sustained ER stress promotes caspase3-dependent apoptosis during the later phase of the ER stress response in NG108-15 cells [1], [2]. In addition, we and others have previously shown that caspase3-mediated cleavage of Syx5 protein accompanies inhibition of secretory traffic during apoptosis [2], [6]. Here, we used a long term real time imaging technique to analyze the effect of various toxins and reagents that affect ER stress on the caspase3/7-dependent apoptosis of neuronal cell line NG108-15 cells.

## 2 Experimental design, materials and methods

**Fig. 1:**
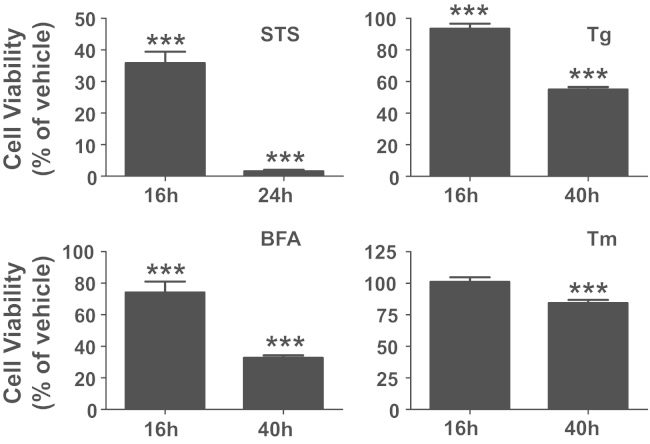
Cell viability of NG108-15 cells treated with apoptosis and ER stress inducers. NG108-15 cells in PLL-coated 96 well plates were treated with vehicle or 0.1 μM STS, 1 μM Tg, 2 μg/mL BFA, and 2 μg/mL Tm, for indicated times representing the early and the late stages of ER stress response. Cell viability assay was performed as described in Section 2. Each value is represented as a mean±S.D. percentage of vehicle control (*n*=8–32, *t*-test, vs*.* vehicle control, ***P<0.0001). STS and ER stressors caused time dependent reduction in cell viability of NG108-15 cells.

**Fig. 2:**
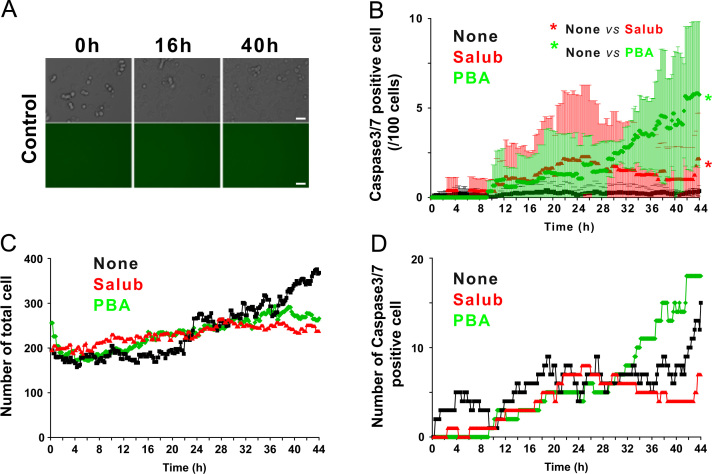
Time lapse imaging of caspase3/7 activation in cells treated with salubrinal and sodium 4-phenylbutyrate. NG108-15 cells were pretreated with CellEvent caspase3/7 indicator prior to the treatment with or without 0.1 mM salubrinal (Salub) or 5 mM sodium 4-phenylbutyrate (PBA), modifiers of ER stress induction. Immediately after the treatment with the fresh medium containing the reagents, NG108-15 cells were subjected to live cell imaging of differential interference contrast (DIC) and green fluorescence images as described in Section 2. Representative images from control cells at 16 h and 40 h after treatment are shown (A). The total number of cells and the number of fluorescence-emitting cells (active caspase3/7 positive) at each time point was counted as described in Section 2. Analyses of the mean number of caspase3/7 positive cells/100 cells (B) number of total cells in each time frame (C), and the total number of caspase3/7 positive cells in each time frame (D) were plotted as a function of time. The population of caspase3/7 dependent apoptotic cells was less than approximately 10% of total cells after 40 h of treatment with Salub or PBA alone in NG108-15 cells. Scale bar, 50 μm.

**Fig. 3:**
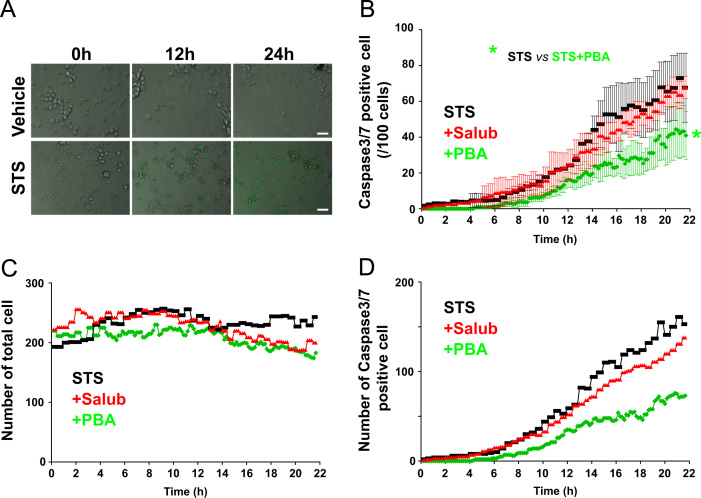
Effects of Salub and PBA on STS-induced activation of caspase3/7. NG108-15 cells were treated with 0.1 mM Salub or 5 mM PBA in the presence of 0.1 μM STS and subjected to time lapse imaging analyses as in Fig. 2. Representative overlayed images of DIC and green fluorescence from vehicle- and STS- treated cells are shown (A). Analyses of the mean number of caspase3/7 positive cells/100 cells (B) number of total cells in each time frame (C), and the total number of caspase3/7 positive cells in each time frame (D) were plotted as a function of time. Activated caspase3/7 dependent apoptosis are markedly induced by STS treatment. Although the ER stress-triggered cell death is not only due to caspase3/7, but compared to the ratio of dead cells obtained from the cell viability assay of STS-treated cells (Fig. 1), it can be estimated that approximately 79% of dead cells are caspase3/7 positive after 16 h of treatment. PBA, but not Salub, significantly alleviated STS-induced caspase3/7 dependent apoptosis. Scale bar, 50 μm.

**Fig. 4:**
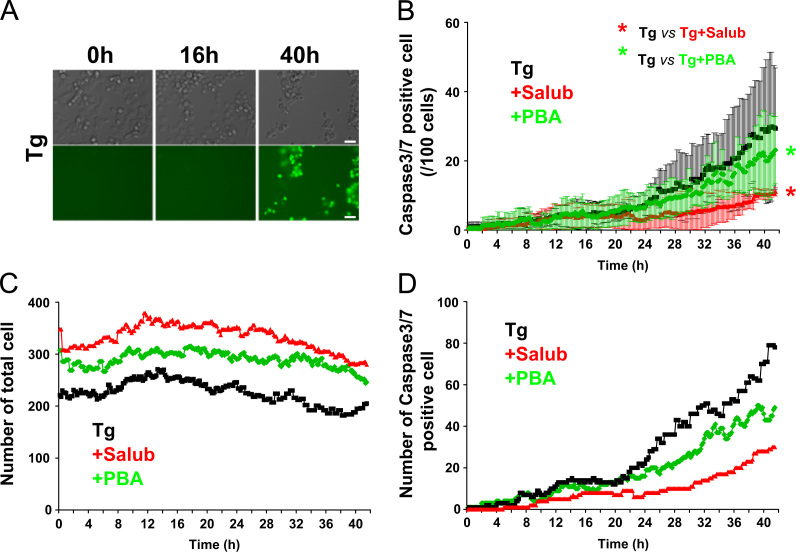
Effects of Salub and PBA on the activation of caspase3/7 treated with thapsigargin (Tg). NG108-15 cells were treated with 0.1 mM Salub or 5 mM PBA in the presence of 1 μM Tg and subjected to time lapse imaging analyses as in Fig. 2. Representative images of DIC and green fluorescence from Tg-treated cells are shown (A). Analyses of the mean number of caspase3/7 positive cells/100 cells (B) number of total cells in each time frame (C), and the total number of caspase3/7 positive cells in each time frame (D) were plotted as a function of time. Apoptosis due to activated caspase3/7 was induced by Tg treatment. Compared to the ratio of dead cells obtained from the cell viability assay of Tg-treated cells (Fig. 1), it can be estimated that approximately 77% and 67% (16 h and 40 h, respectively) of dead cells are caspase3/7 positive. Both Salub and PBA, significantly alleviated Tg-induced caspase3/7 dependent apoptosis. Scale bar, 50 μm.

**Fig. 5:**
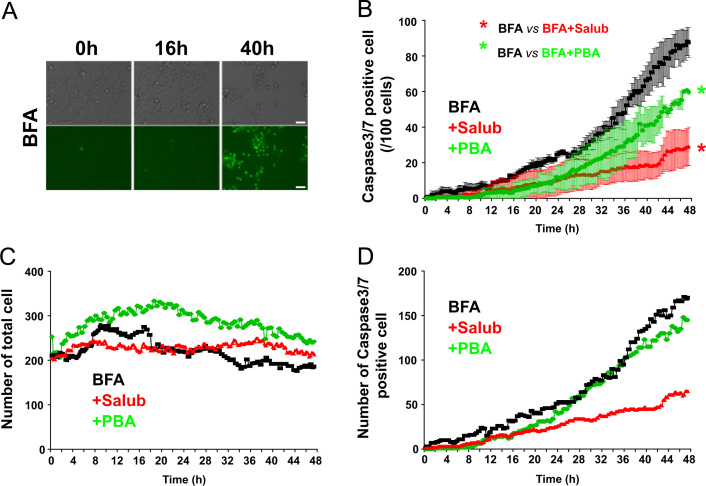
Effects of Salub and PBA on the activation of caspase3/7 treated with Brefeldin A (BFA). NG108-15 cells were treated with 0.1 mM Salub or 5 mM PBA in the presence of 2 μg/mL BFA and subjected to time lapse imaging analyses as in Fig. 4. Representative images of DIC and green fluorescence from BFA-treated cells are shown (A). Analyses of the mean number of caspase3/7 positive cells/100 cells (B) number of total cells in each time frame (C), and the total number of caspase3/7 positive cells in each time frame (D) were plotted as a function of time. Apoptosis due to activated caspase3/7 was markedly induced by BFA treatment. Compared to the ratio of dead cells obtained from the cell viability assay of BFA-treated cells (Fig. 1), it can be estimated that approximately 58% and 97% (16 h and 40 h, respectively) of dead cells are caspase3/7 positive. Both Salub and PBA significantly alleviated BFA-induced caspase3/7 dependent apoptosis. Scale bar, 50 μm.

**Fig. 6:**
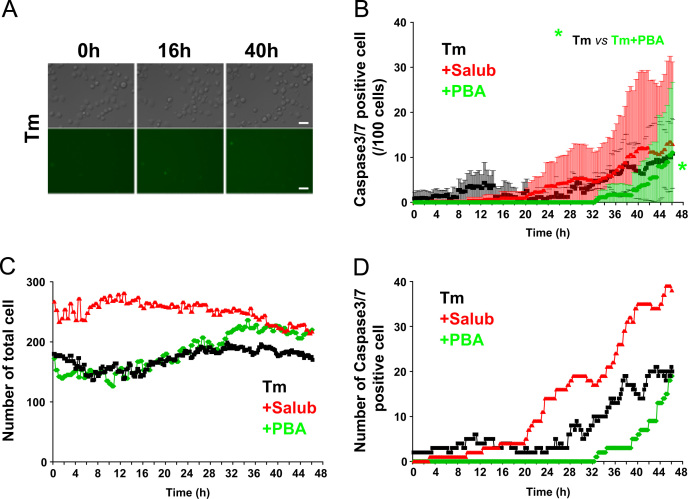
Effects of Salub and PBA on the activation of caspase3/7 treated with Tunicamycin (Tm). NG108-15 cells were treated with 0.1 mM Salub or 5 mM PBA in the presence of 2 μg/mL Tm and subjected to time lapse imaging analyses as in Fig. 4. Representative images of DIC and green fluorescence from Tm-treated cells are shown (A). Analyses of the mean number of caspase3/7 positive cells/100 cells (B) number of total cells in each time frame (C), and the total number of caspase3/7 positive cells in each time frame (D) were plotted as a function of time. PBA, but not Salub, alleviated Tm-induced caspase3/7 dependent apoptosis. Scale bar, 50 μm.

We first examined the cell viability of NG108-15 cells treated with the strong apoptosis inducer Staurosporine (STS) and various toxins that cause ER stress (Fig. 1). Apoptotic cells were defined by the activation of the caspase3/7 fluorescent reporter, which was assessed by the appearance of green fluorescence emission in the cells. Induction of apoptosis by STS caused prominent activation of caspase3/7, whereas vehicle treated control cells show no fluorescence after 24 h of treatment (Fig. 3). We utilized two different ER stress modulators (Fig. 2). One is Salubrinal (Salub) that has been shown to protect cell from ER stress [7], and the other is a chemical chaperone sodium 4-phenylbutyrate (PBA) that has been reported to rescue the proteolytic deficit [8]. We first examined the effects of ER stress modulating reagents Salub and PBA on ER stress-induced apoptosis using time lapse imaging (Fig. 2). We examined their effects on STS-induced apoptosis in NG108-15 cells and showed the different effects of these two modulators (Fig. 3). In order to see their effects on ER stress-induced apoptosis triggered by ER stress, we used three different types of toxins for the ER stressors. Treatment of NG108-15 cells with Thapsigargin (Tg), a potent ER stress-inducing toxin that perturbs ER Ca^2+^ homeostasis by inhibiting sarco/endoplasmic reticulum Ca^2+^-ATPase, caused gradual increase in apoptotic cells after 24 h of treatment (Fig. 4). We also treated cells with the fungal toxin Brefeldin A (BFA) (Fig. 5), which is known to induce accumulation of proteins in the ER due to inhibition of protein trafficking through the early secretory compartments [9]. Cells were also treated with Tunicamycin (Tm), a toxin that inhibits *N*-linked glycosylation of proteins in the ER (Fig. 6). Data showing the difference in the action between Salub and PBA on the alleviation of the caspase3/7 dependent apoptosis induced by various toxins and reagents are presented (Fig. 3, Fig. 4, Fig. 5, Fig. 6).

### 2.1 Materials

Tm and BFA were purchased from Sigma-Aldrich Chemical Co. (St. Louis, MO). Salb was purchased from Enzo (Farmingdale, NY). STS, Tg, and PBA were purchased from Merck (Darmstadt, Germany). All other reagents were of the highest grade available, unless otherwise noted.

### 2.2 Cell culture

Mouse neuroblastoma and rat glioma hybrid NG108-15 cells were cultured in Dulbecco׳s modified Eagle׳s medium containing 4 mM l-glutamine, 100 U/mL of penicillin, 100 µg/mL of streptomycin, 2.5 mM hypoxanthine, 10 µM aminopterin, 0.4 mM thymidine, and 10% fetal bovine serum in a humidified incubator with 5% CO_2_ at 37 °C, as described previously [10]. The cells were inoculated into poly-l-lysine (PLL)-coated 35-mm dishes (BD Bioscience) with glass bottoms for time lapse imaging analyses.

### 2.3 Real-time imaging of caspase 3/7 activation

NG108-15 cells were plated in glass-bottomed 35-mm dishes coated with PLL (Sigma-Aldrich) prior to the day of imaging. Cells were incubated in culture medium containing CellEvent caspase3/7 reagent (Life Technologies, Rockland, MD, USA) for 30 min in a humidified chamber maintained at 5% CO_2_ and 37 °C. After incubation, 2 mL of fresh medium was added and the dish was placed in an imaging chamber kept under the same conditions as the CO_2_ incubator using a stage-top chamber equipped with a digital gas mixer (GM-8000, TokaiHit, Shizuoka, Japan). In the early frames at the beginning of real time imaging, rounding of the cell is observed due to the stimuli of changing to the fresh medium done just before the starting of time lapse imaging. The rounding of cells during the cell cycle can be also observed before cell division. Digital images of stained cells were acquired using an IX81 inverted microscope (Olympus) equipped with a 20× UPLSApo lens (numerical aperture, 0.75) and a CoolSNAP-HQ CCD camera (Roper Scientific) using appropriate filters for emission. LED (CoolLED) was used for excitation. Images were acquired every 10 min and collected as TIFF files using MetaMorph software (Universal Imaging). Whole number of cells and caspase3/7 positive cells were counted within the area of the acquired image (450×335 µm^2^) from each time point. Apoptotic cells were defined by the activation of the caspase3/7 fluorescent reporter, which was assessed by the appearance of green fluorescence emission in the cells. This manual counting was conducted by a blinded examiner. The number of all of the cells and caspase3/7 positive cells present in each frame were counted. In addition, 100 cells that were randomly selected from the frame in each time points were also subjected to counting of caspase3/7 positive cells. The values are expressed as mean number of positive cells/100 cells obtained from at least two different experiments. Total number of cells varies from frame to frame since NG108-15 cells are adhered to the coverslips, but very mobile and also proliferate during long term imaging. Very few cells may detach, however, we cannot discriminate between detached cells and cells that were migrated out of the imaging frame. Although the morphology is changed, cells that underwent cell death are still attached (as a cell debris) to the coverslip because the coverslips are coated with PLL and the indicator dye for caspase3/7 activation binds to DNA in the nuclei when cleaved by caspase3/7. These features allowed us to measure the number of dead cells throughout the consecutive imaging acquisition even after cells have undergone apoptotic cell death. The number of all the caspase3/7 positive cells at each time point, the total number of cells at each time point, and the number of caspase3/7 positive cells per 100 cells, were plotted as a function of time.

### 2.4 Cell viability assay

Cell viability was measured with a CellTiter-Glo Luminescent Cell Viability Assay system (Promega) according to the manufacturer׳s instructions. Briefly, cells were harvested in PLL-coated 96-well plates (Greiner Bio-One) and treated with various reagents. Subsequently, the intracellular ATP levels were determined by a bioluminescence reaction using thermostable recombinant firefly luciferase and beetle luciferin. The luminescence was measured using a GloMax Luminometer (Promega). The level of luminescence produced is proportional to the cell number present in each well.

### 2.5 Data analysis and statistics

Data are presented as mean±SEM where otherwise noted, with ‘*n*’ indicating the number of samples examined. For the cell viability analysis, a *t*-test was used to determine the statistical significance of differences between values. Data sets from time-lapse imaging were subjected to one-way ANOVA and Bonferroni multiple comparisons analysis with post-hoc tests. *P<0.05.
